# Metabolomics profiling to characterize cerebral ischemia-reperfusion injury in mice

**DOI:** 10.3389/fphar.2023.1091616

**Published:** 2023-02-06

**Authors:** Qiong Chen, Ting Zhou, Jun-jie Yuan, Xiao-yi Xiong, Xue-hui Liu, Zong-ming Qiu, Lin-lin Hu, Hui Lu, Qian He, Chang Liu, Qing-wu Yang

**Affiliations:** ^1^ Department of Neurology, Xinqiao Hospital, The Army Medical University (Third Military Medical University), Chongqing, China; ^2^ Sichuan Provincial Key Laboratory for Acupuncture & Chronobiology, Acupuncture and Tuina School, Chengdu University of Traditional Chinese Medicine, Chengdu, China; ^3^ School of Basic Medical Sciences, Chengdu University of Traditional Chinese Medicine, Chengdu, China; ^4^ Department of Medicinal Chemistry, College of Pharmacy, The Army Medical University (Third Military Medical University), Chongqing, China

**Keywords:** ischemic-reperfusion, metabolites, non-targeted metabolomics, UPLC/Q-TOF-MS, GC-TOF-MS

## Abstract

Cerebral ischemia, resulting from compromised blood flow, is one of the leading causes of death worldwide with limited therapeutic options. Potential deleterious injuries resulting from reperfusion therapies remain a clinical challenge for physicians. This study aimed to explore the metabolomic alterations during ischemia-reperfusion injury by employing metabolomic analysis coupled with gas chromatography time-of-flight mass spectrometry (GC-TOF-MS) and ultraperformance liquid chromatography quadrupole (UPLC/Q)-TOF-MS. Metabolomic data from mice subjected to middle cerebral artery occlusion (MCAO) followed by reperfusion (MCAO/R) were compared to those of the sham and MCAO groups. A total of 82 simultaneously differentially expressed metabolites were identified among each group. The top three major classifications of these differentially expressed metabolites were organic acids, lipids, and organooxygen compounds. Metabolomics pathway analysis was conducted to identify the underlying pathways implicated in MCAO/R. Based on impactor scores, the most significant pathways involved in the response to the reperfusion after cerebral ischemia were glycerophospholipid metabolism, linoleic acid metabolism, pyrimidine metabolism, and galactose metabolism. 17 of those 82 metabolites were greatly elevated in the MCAO/Reperfusion group, when compared to those in the sham and MCAO groups. Among those metabolites, glucose-6-phosphate 1, fructose-6-phosphate, cellobiose 2, o-phosphonothreonine 1, and salicin were the top five elevated metabolites in MCAO/R group, compared with the MCAO group. Glycolysis, the pentose phosphate pathway, starch and sucrose metabolism, and fructose and mannose degradation were the top four ranked pathways according to metabolite set enrichment analysis (MSEA). The present study not only advances our understanding of metabolomic changes among animals in the sham and cerebral ischemia groups with or without reperfusion *via* metabolomic profiling, but also paves the way to explore potential molecular mechanisms underlying metabolic alteration induced by cerebral ischemia-reperfusion.

## 1 Introduction

Cerebral ischemia, resulting from compromised blood flow, is one of the leading causes of death worldwide with limited therapeutic options (DeSai and Hays Shapshak, 2022). Acute reperfusion therapies, including intravenous thrombolytics and mechanical thrombectomy, which can help with the restoration of cerebral blood flow and energy supply, have been used for the treatment of acute ischemic stroke (Imran et al., 2021). However, the clinical outcomes after reperfusion to ischemic brain are not optimal, as expected. Researchers have demonstrated that the restoration of oxygen and glucose supply resulting from reperfusion could also cause subsequent deleterious injury aside from ischemia alone, eventually leading to profound inflammatory and neuronal death in the brain (Wu et al., 2018). Advanced understanding of ischemia-reperfusion (IR) injury revealed that several underlying pathophysiological mechanisms are involved in reperfusion-induced injuries, such as oxidative stress (de Vries et al., 2013; Wu et al., 2020; 2016), excitotoxicity, mitochondrial dysfunction, activation of the complement system, blood-brain-barrier disruption, and neuroinflammation (2016). Owing to the above-mentioned complexity of IR injury, no effective therapeutic targets have been developed to date. Therefore, further investigations of potential molecular pathways and neuroprotective interventions to minimize reperfusion injury are urgently warranted to improve the clinical outcomes of patients with ischemic stroke.

The brain requires 20%–25% of the energy provided by basal metabolism, which is a massive demand for energy considering its relatively small size and weight (Camandola and Mattson, 2017). Energy metabolism in the brain plays a critical role in the maintenance of functionality of the central nervous system, which is highly depended on the blood flow and supply of glucose and oxygens to neurons and glial cells (Watts et al., 2018). Cerebral ischemia-induced disruption of oxygen consumption and glucose utilization contributes to metabolic perturbations of the brain, while reperfusion injury leads to further metabolic alterations and increased brain infarct volume compared to permanent occlusion (Zhang et al., 1994). However, metabolic profiles after reperfusion are poorly characterized.

Advances in omics have allowed for comprehensive and systemic profiling of small molecular substances alterations, which provided scientists with comprehensive insight into mechanisms underlying the pathogenesis of diseases. Metabolomic analysis can be used to identify potential pathways and understand pathological mechanisms (Wang et al., 2014; Shin et al., 2020). In this study, we employed metabolomic analysis coupled with gas chromatography time-of-flight mass spectrometry (GC-TOF-MS) and ultraperformance liquid chromatography quadrupole (UPLC/Q)-TOF-MS to determine the alterations in the brain metabolome of mice after 90 min of middle cerebral artery occlusion (MCAO) with or without reperfusion for 24 h. This study aimed to investigate the metabolic characteristics and pathogenesis of cerebral ischemia-reperfusion injury to shed a light on therapeutic interventions for cerebral ischemia-reperfusion injury.

## 2 Materials and methods

### 2.1 Animals

Adult male C57BL/6 mice (specific-pathogen-free, eight-week-old, 20–25 g) were obtained from the Animal Center of the Army Medical University (Chongqing, China; Certificate No. SCXK 2019–0004). Colonies were maintained in a specific pathogen-free grade environment with a 12 h light/12 h dark cycle. The use of mice was performed in accordance with the Guide of Care and Use of Experimental Animals of the Animal Ethics Committee of the Army Medical University.

### 2.2 MCAO and MCAO/R models

**FIGURE 1:**
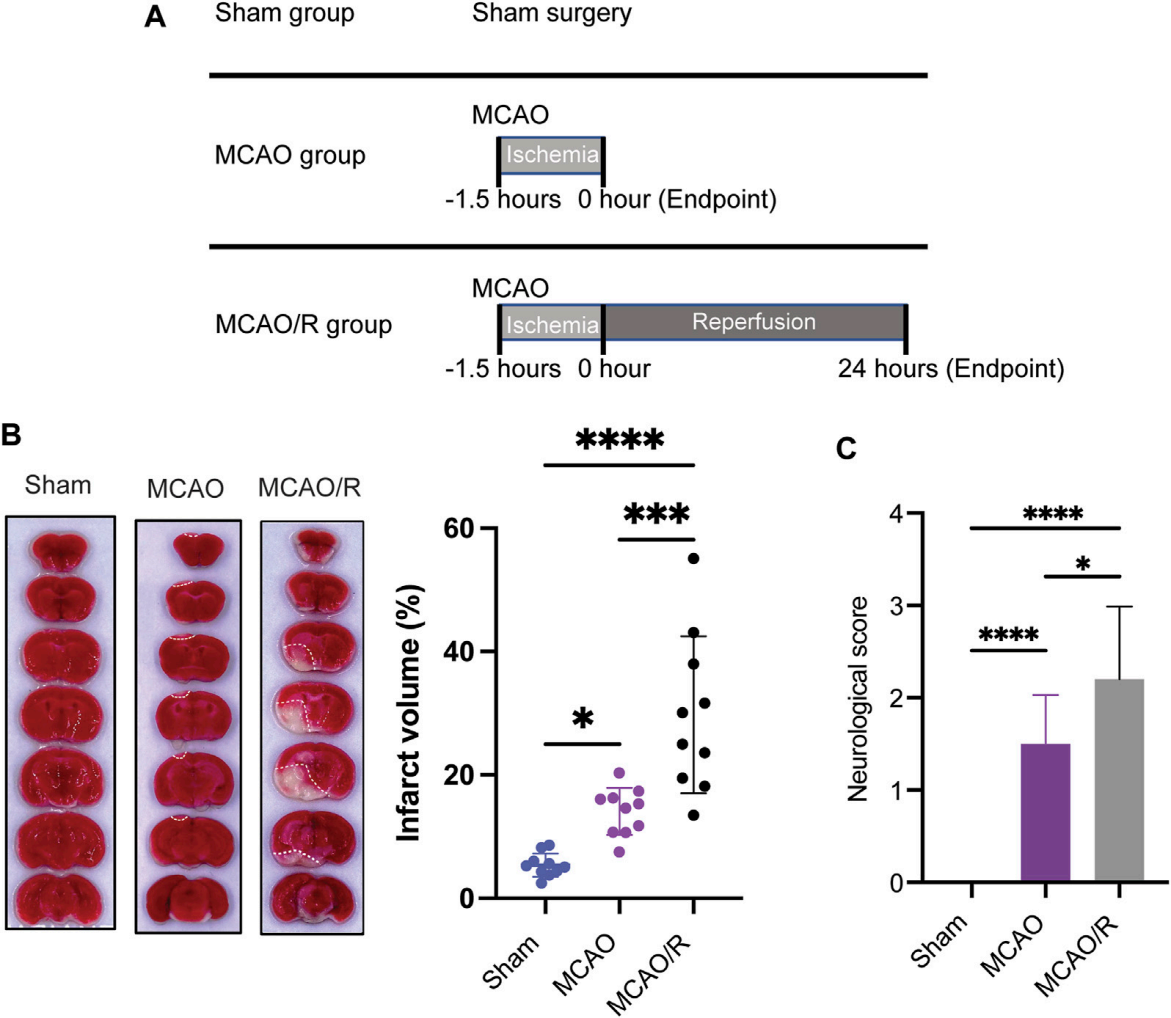
**(A)** Experimental design diagram. Mice were maintained in the animal center for 1 week after arrival for adaptation. Middle cerebral artery occlusion was performed on the eighth day. Reperfusion (for 24 h) was conducted after ischemia (for 1.5 h). Brain tissue was collected after euthanizing the animals. **(B)** Representative of triphenyl tetrazolium chloride (TTC)-stained brain slices and infarct volume measured using the ImageJ software. **(C)** Neurological scores were measured by using a four-point system. N = 10 biological replicates for each group. ****p < 0.0001.

As shown in the flow chart in Figure 1A, the mice from the original mouse cages were randomly allocated to the different experimental groups (sham, MCAO, and MCAO/R; n = 10 in each group). The cerebral ischemia was induced using an intraluminal filament as previously described (Longa et al., 1989). Briefly, the left middle cerebral artery was occluded with a blunt-tip 6–0 nylon monofilament, 16–17 mm past the carotid bifurcation until a slight resistance was felt. Body temperature was maintained at 37°C ± 0.5°C. The MCAO mice were sacrificed immediately after 1.5 h of ischemia, while the mice in the MCAO/R group were subsequently reperfused for 24 h by the careful withdrawal of the filament. The MCAO/R mice were euthanized after 24 h of reperfusion. Animals in the sham group were subjected to sham surgery, which underwent the same procedure but without insertion of filament to occlude the carotid bifurcation. Animals were anesthetized and transcardially perfused with 20 mL of phosphate-buffered saline (PBS). Brain tissues were collected for the fsubsequent analyses.

### 2.3 Infarct volume measurement and neuroscore assessment

Infarct volume measurements and neuroscore assessments were performed according to a previously developed method (Longa et al., 1989). Brain tissues were rapidly removed, frozen at −20°C for 15 mins, and coronally sectioned into 1–2 mm slices from the frontal tips. Sections were stained with 2% 2,3,5-tripenyltetrazolium chloride (TTC, Sangon biotech, Shanghai) at 37°C for 30 mins, and then stored in 4% paraformaldehyde. The infarct volume (white or pale pink areas) was measured as a percentage of the total brain volume using the ImageJ software.

Neuroscore assessment was performed by an experimenter blinded to the experimental groups (Rating scale: 0 = no deficit, 1 = failure to extent the left forepaw, 2 = decreased grip strength of the left forepaw, 3 = circling to the left by pulling the tail, and 4 = spontaneous circling).

### 2.4 Metabolite extraction

For LC with tandem mass spectrometry (LC-MS/MS) detection, metabolite extraction was performed according to a previously developed method (Rashad et al., 2020). Fifty milligrams of sample were weighted in an EP tube, and 1,000 μL extract solution (acetonitrile: methanol: water = 2:2:1) with 1 μg/mL internal standard was added. After 30 s vortex, the samples were homogenized at 35 Hz for 4 min and sonicated for 5 min on ice. Homogenization and sonication cycles were repeated for three times. Then the samples were incubated for 1 h at −40°C and centrifuged at 10000 rpm for 15 min at 4°C. The resulting supernatant was transferred to a fresh glass vial for analysis. The quality control (QC) sample was prepared by mixing an equal aliquot of the supernatants from all the samples.

For GC/TOF-MS analysis, metabolite extraction was performed according to a previously developed method (Wang et al., 2017). A 50 ± 1 mg sample was transferred into a 2 mL tube, and 1,000 μL pre-cooled extraction mixture (methanol/chloroform (v:v) = 3:1) with 0.5 μg/mL internal standard was added. Each sample prepared with the same procedure as in LC-MC/MC was evaporated in a vacuum concentrator. Then, 40 μL of methoxyamination hydrochloride (20 mg/mL in pyridine) was added and the sample was subsequently incubated at 80°C for 30 mins, then derivatized with 60 μL of BSTFA regent (1% TMCS, v/v) at 70°C for 1.5 h. Next, samples were gradually cooled to room temperature, and 5 μL of FAMEs (in chloroform) was added to the QC sample. All samples were then analyzed using GC-TOF-MS.

### 2.5 LC-MS/MS analysis

LC-MS/MS analyses were performed using an UHPLC system (1,290, Agilent Technologies) with a UPLC HSS T3 column (2.1 mm × 100 mm, 1.8 μm) coupled to a Q Exactive mass spectrometer (Orbitrap MS, Thermo) according to a previously developed method (Rashad et al., 2020). Mobile phase A consisted of 0.1% formic acid in water for the positive mode, and 5 mmol/L ammonium acetate in water for the negative mode. Mobile phase B consisted of acetonitrile. The elution gradient was set as follows: 0–1.0 min, 1% B; 1.0–8.0 mins, 1%–99% B; 8.0–10.0 mins, 99% B; 10.0–10.1 mins, 99%–1% B; and 10.1–12 mins, 1% B. The flow rate was 0.5 mL/min. The injected volume was 3 μL. The QE mass spectrometer was used for its ability to acquire MS/MS spectra on information-dependent acquisition (IDA) mode in the sham of the acquisition software (Xcalibur 4.0.27, Thermo). In this mode, the acquisition software continuously evaluated the full-scan MS spectrum. The electrospray ionization source conditions were set as follows: sheath gas flow rate of 45 Arb, Aux gas flow rate of 15Arb, capillary temperature of 400°C, full MS resolution of 70000, MS/MS resolution of 17500, collision energy of 20/40/60 eV in normalized collision energy mode, and spray Voltage of 4.0 kV (positive) or −3.6 kV (negative), respectively.

### 2.6 GC-TOF-MS analysis

GC-TOF-MS analysis was performed using an Agilent 7,890 gas chromatograph coupled with a TOF mass spectrometer according to a previously developed method (Fiehn, 2016). The system utilized a DB-5MS capillary column. A 1 μL aliquot of the sample was injected in splitless mode. Helium was used as the carrier gas, the front inlet purge flow rate was 3 mL/min, and the gas flow rate through the column was 1 mL/min. The initial temperature was maintained at 50°C for 1 min, then raised to 310°C at a rate of 10°C/min, and then maintained for 8 min at 310°C. The injection, transfer line, and ion source temperatures were 280, 280°Cand 250°C, respectively. The energy was −70 eV in the electron impact mode. Mass spectrometry data were acquired in full-scan mode with the m/z range of 50–500 at a rate of 12.5 spectra per second after a solvent delay of 6.25 min.

### 2.7 Metabolome data processing

Raw data from the LC-MS/MS analysis were converted to the mzXML format using ProteoWizard and processed using an in-house program, which was developed using R and based on XCMS, for peak detection, extraction, alignment, and integration according to a previously described method (Smith et al., 2006). An in-house MS2 database (BiotreeDB) was usded for metabolite annotation. The cutoff for annotation was set at 0.3.

Raw data from the GC-TOF-MS analysis, including peak extraction, baseline adjustment, deconvolution, alignment and integration, were completed using the Chroma TOF (V 4.3x, LECO) software. The LECO-Fiehn Rtx5 database was used for metabolite identification by matching the mass spectrum and retention index according to a previously described study (Dunn et al., 2011). Finally, the peaks detected in less than half of the QC samples or those with RSD>30% in the QC samples were removed.

### 2.8 Statistical analysis

Differences among multiple groups were determined using one-way ANOVA, followed by Bonferroni’s multiple comparisons test. Statistical analyses were performed using the GraphPad software (GraphPad Prism, United States). Data are presented as the mean ± SD. A *p*-value of <0.05 was considered statistically significant.

## 3 Results

### 3.1 Reperfusion increased the infarct volume following focal ischemia

To examine and compare the ischemic brain damage induced by MCAO with or without reperfusion, TTC (2,3,4-triphenyltetrazolium chloride) staining was applied to analyze the infarct size in the sham, MCAO, and MCAO/R groups. Transient MCAO for 90 min followed by 24 h-reperfusion induced a significant increase in the infarct volume and neurological deficit compared with those in the sham and MCAO groups (*p* < 0.05) (Figures 1B, C). Notably, the infarct volume induced by permanent MCAO was greatly smaller than that in MCAO/R group (Figure 1B).

### 3.2 Ischemia and ischemia/reperfusion induced prominent metabolic alterations in the brain

**FIGURE 2:**
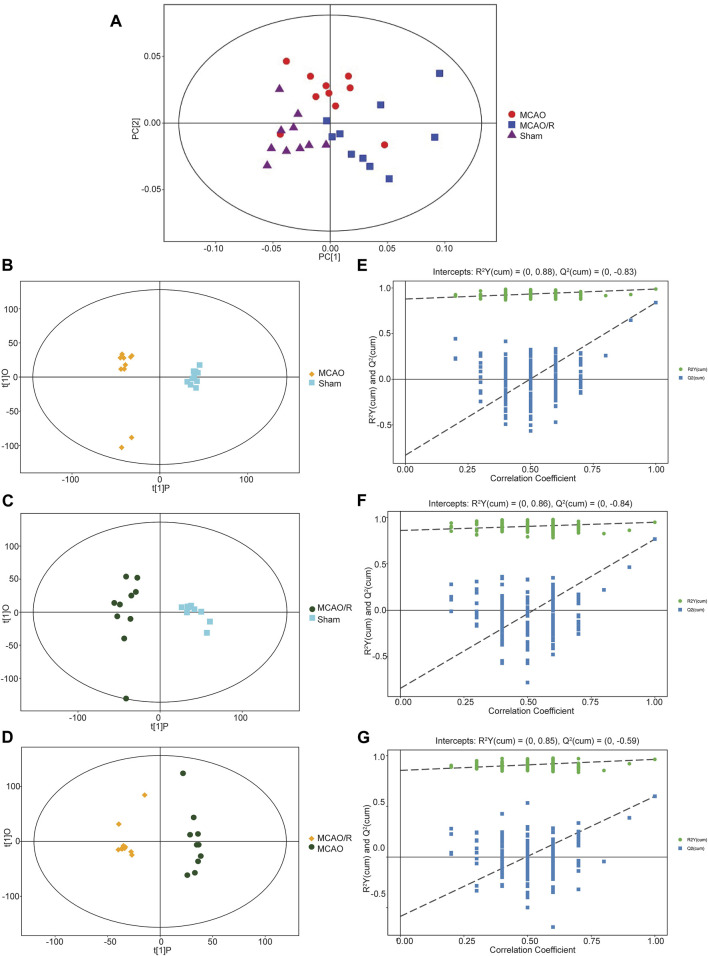
**(A)** Principal component analysis (PCA) plot of samples in the sham group (purple triangle), middle cerebral artery occlusion (MCAO) (red cycle), and MCAO/reperfusion (R) (blue rectangle) groups. N = 10 biological replicates for each group. **(B–G)** Score scatter plot of the orthogonal projections to latent structures-discriminant analysis (OPLS-DA) model for groups of the **(B)** MCAO versus sham, **(C)** MCAO/R versus sham, and **(D)** MCAO/R versus MCAO groups. Permutation test of the OPLS-DA model for the **(E)** MCAO versus sham, **(F)** MCAO/R versus sham, and **(G)** MCAO/R versus MCAO groups.

Multivariate statistical analysis methods, including principal component analysis (PCA) and partial least squares-discriminant analysis (PLS-DA), were employed to investigate the separation of the gas-liquid chromatography data of the sham, MCAO, and MCAO/R groups. As shown in Figure 2A, the PCA plot revealed a clear separation and a close association in the metabolic profiles among the sham group (purple triangle), MCAO (red cycle), and MCAO/R (blue rectangle) groups. Consistent with the PCA plot, the PLS-DA plots of the MCAO versus sham, MCAO/R versus sham, and MCAO/R versus MCAO groups were cross-validated, demonstrating the metabolic differences among these three groups (Figures 2B–D). In addition, the permutation plots indicated that the original PLS-DA model was valid and had no overfitting (Figures 2E–G).

### 3.3 Differential metabolomic profiling among the sham, MCAO, and MCAO/R samples

**FIGURE 3:**
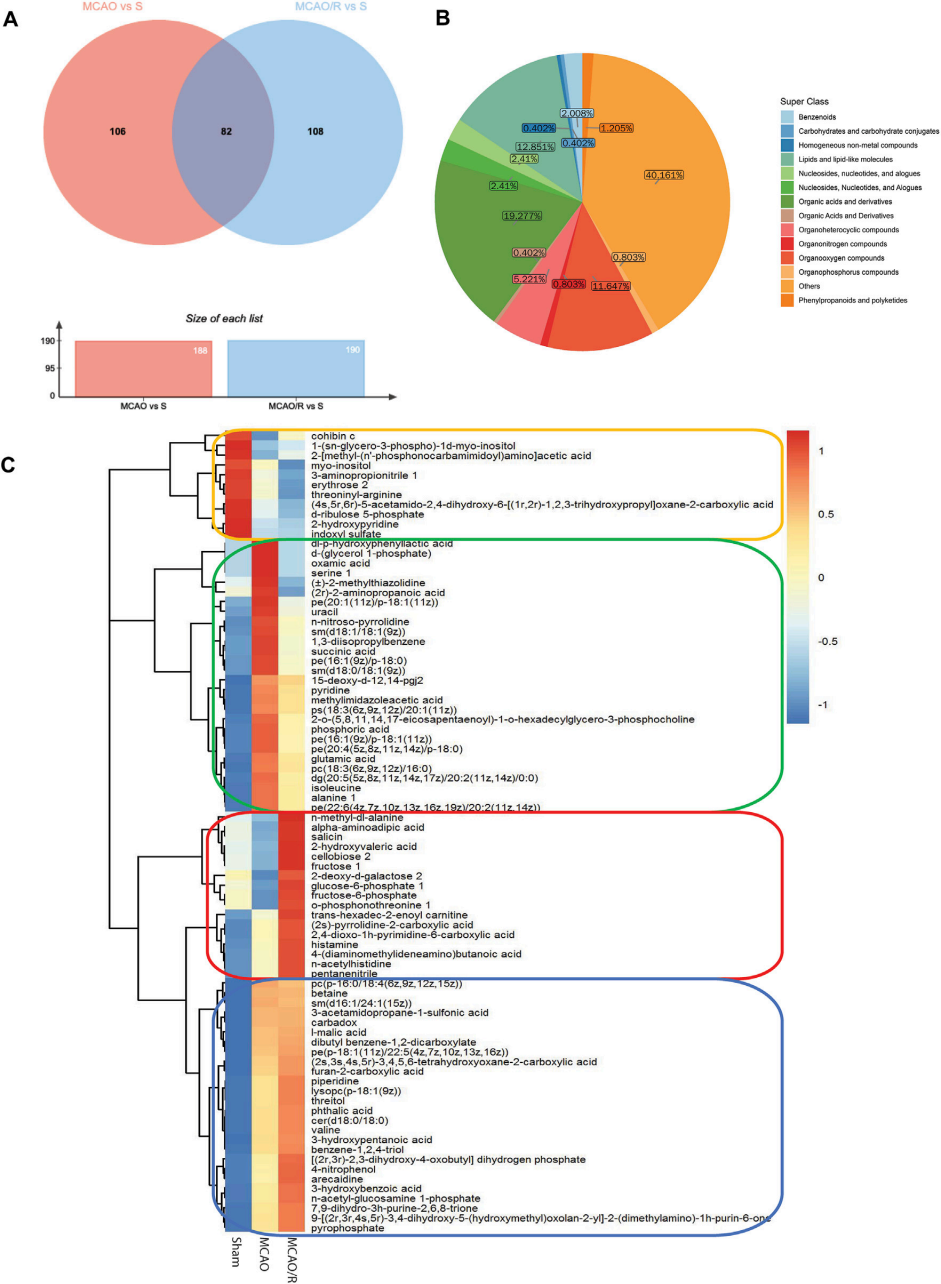
**(A)** Venn diagram of differential metabolites between groups the middle cerebral artery occlusion (MCAO) versus sham and MCAO/reperfusion (R) versus sham groups. **(B)** Metabolic categories of the 82 identified metabolites. **(C)** Heatmap profile of the 82 metabolites that are simultaneously differentially expressed in the sham, MCAO, and MCAO/R groups. Red and blue indicate upregulation and downregulation relative to the median level, respectively (see color scale).

Differential metabolomic profiling analysis was performed to identify the correlated metabolites involved in the reperfusion-induced alterations. A total of 188 differentially expressed metabolites were identified in the sham versus MCAO group, while 190 metabolites were significantly altered in the MCAO/R versus sham group. A total of 82 metabolites were simultaneously differentially expressed in these three groups, according to the Venn diagram (Figure 3A). The category showed that 19.27%, 12.851%, and 11.647% of the metabolites belonged to organic acids, lipids, and organooxygen compounds, respectively (Figure 3B). In addition, the changes in the levels of the 82 identified metabolites were plotted using a hierarchical clustering heatmap (Figure 3C). Specifically, the levels of 28 metabolites were significantly upregulated after MCAO and returned to near baseline levels after reperfusion (green box, Figure 3C), while the levels of 26 metabolites in both the MCAO and MCAO/R groups were elevated relative to those in the sham group (blue box, Figure 3C). In addition, the levels of 11 metabolites in both the MCAO and MCAO/R groups were reduced when compared with those in the sham group (orange box, Figure 3C). Notably, the levels of 17 out of these 82 differential metabolites in the MCAO/R group were significantly elevated when compared with those in the sham and MCAO groups (red box, Figure 3C).

### 3.4 Metabolic pathway analysis (MetPA) of differential metabolites in the response to MCAO or MCAO/R

**FIGURE 4:**
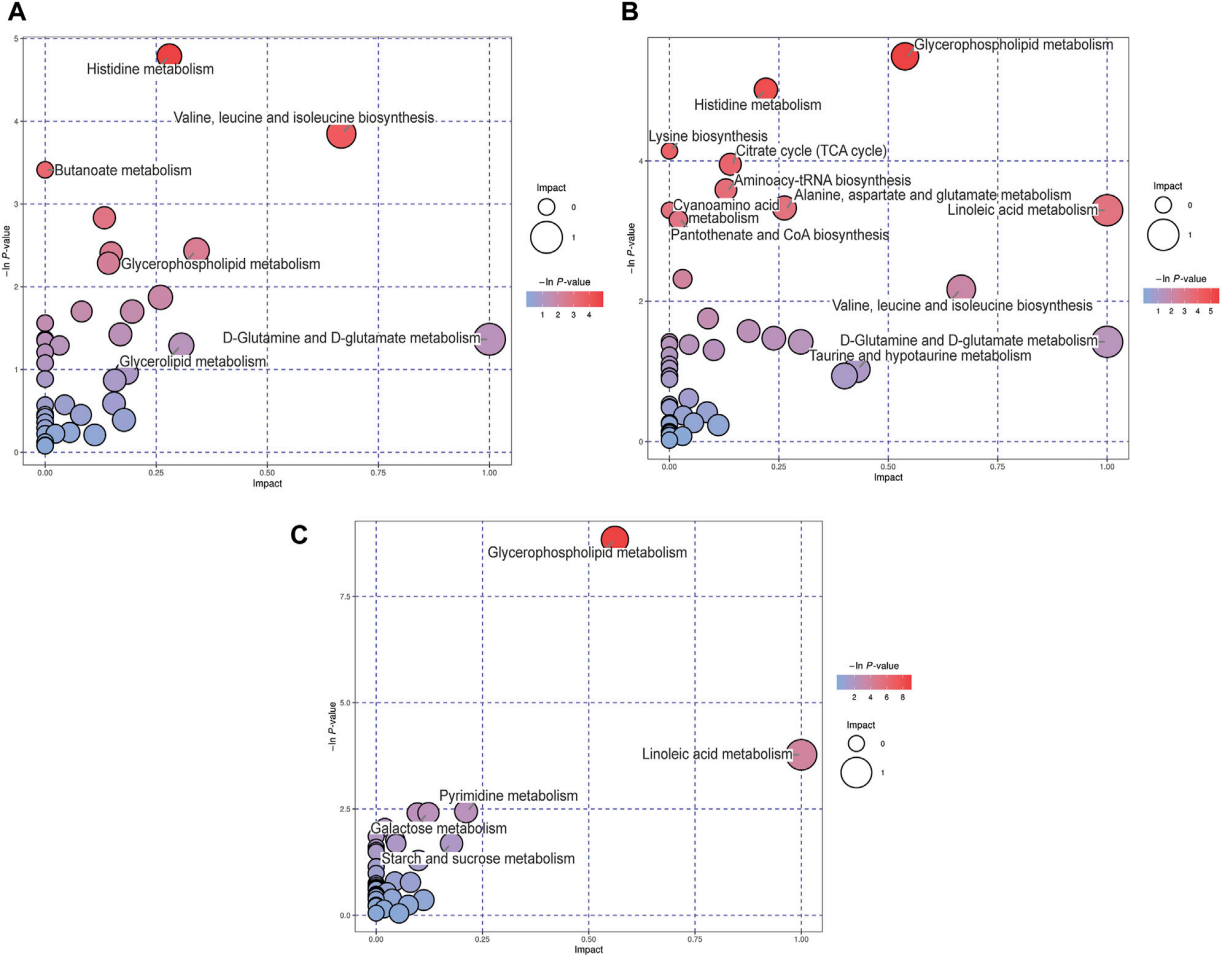
Metabolic pathway analysis (MetPA) analysis of differential metabolites for the **(A)** middle cerebral artery occlusion (MCAO) versus sham, **(B)** MCAO/reperfusion (R) versus sham, and **(C)** MCAO/R versus MCAO groups. The -ln *p*-value on the y-axis and the size of each circle were obtained from the pathway enrichment analysis, while the impact values on the x-axis were from the pathway topology analysis.

Differential metabolites among these groups were selected and subjected to MetPA to identify the metabolic pathways involved. “The metabolome view” showed that multiple pathways, including histidine metabolism, butanoate metabolism, valine, leucine and isoleucine biosynthesis, glycerophospholipid metabolism, and D-glutamine and D-glutamate metabolism pathways were the most significantly altered in the MCAO group, compared to the sham group, according to their impact value (x-axis) or -ln *p*) value (y-axis) (Figure 4A). In addition, the following pathways: glycerophospholipid metabolism, histidine metabolism, citrate cycle, aminoacyl-tRNA biosynthesis, alanine, aspartate and glutamate metabolism, and linoleic acid metabolism were significantly altered in the MCAO/R group, compared to the sham group (Figure 4B). Relative to MCAO, reperfusion induced alterations in pathways associated with glycerophospholipid metabolism, linoleic acid metabolism, pyrimidine metabolism, and galactose metabolism (Figure 4C).

### 3.5 Identified metabolites and involved pathways involved in reperfusion response

We focused on the reperfusion-induced alterations in the levels of metabolites and found that concentrations of the 17 identified metabolites (red box in Figure 3C) in the MCAO/R group were higher than those in both the sham and MCAO groups. As shown in Table 1, among these metabolites, glucose-6-phosphate 1 (G6P, PubChem CID 439958), fructose-6-phosphate (F6P, PubChem CID 69507), cellobiose 2 (PubChem CID 294), o-phosphonothreonine 1 (PubChem CID 3246323), and salicin (PubChem CID 439503) were the top five elevated metabolites in the group of MCAO/R versus MCAO. In addition, the level of G6P with 50.75 of fold change showed the greatest change in the MCAO/R versus MCAO groups (Table 1A). Moreover, amino acids, including N-acetylhistidine (PubChem CID 75619) and N-methyl-dl-alanine (PubChem CID 4377), and the organooxygen compounds cellobiose 2 (PubChem CID 294) were identified as the top three up-regulated metabolites in the MCAO/R group when compared with those in the sham group (Table 1B).

**TABLE 1:** List of upregulated metabolites (red box in Figure 3C) in mouse brain samples in the (A) middle cerebral artery occlusion/reperfusion (MCAO/R) versus MCAO group and (B) MCAO/R versus sham group. VIP, variable importance in the projection. ***p < 0.001, **p < 0.01, *p < 0.05.

(A)							
No.	Peak	PubChem CID	VIP	*p*-value	Fold change	LOG_2_-FOLD change	Symbol
1	Glucose-6-phosphate 1	439958	2.864225757	0.000804747	50.74652331	5.66523708	***
2	Fructose-6-phosphate	69507	2.765175119	0.000806093	46.90199453	5.55157737	***
3	Cellobiose 2	294	2.804271305	0.000987392	19.73237667	4.302492827	***
4	O-phosphonothreonine 1	3246323	2.721198038	1.00348E-05	14.21486503	3.829328495	***
5	Salicin	439503	2.548609014	0.008142101	7.884287086	2.978980309	**
6	N-methyl-dl-alanine	4377	2.676090698	0.002328932	6.995360732	2.806398455	**
7	Fructose 1	2723872	2.599885584	7.17725E-05	3.894194913	1.961325096	***
8	Alpha-aminoadipic acid	469	2.365301057	0.002195341	3.171746923	1.665277662	**
9	2-deoxy-d-galactose 2	439804	2.845734446	6.11E-08	2.276842157	1.187034279	***
10	2-hydroxyvaleric acid	98009	2.671278079	4.87E-06	1.957959376	0.969350832	***
11	N-acetylhistidine	75619	0.438649611	0.271502312	1.683982608	0.751877239	
12	Pentanenitrile	8061	0.883952465	0.245370607	1.475431114	0.561136565	
13	Histamine	774	0.835540146	0.171526856	1.453719767	0.539749189	
14	Trans-hexadec-2-enoyl carnitine	53477817	1.586571196	0.055386681	1.452087929	0.538128816	
15	4-(diaminomethylideneamino)butanoic acid	500	1.203379571	0.115068444	1.316114929	0.396285477	
16	(2s)-pyrrolidine-2-carboxylic acid	145742	1.003407193	0.217092683	1.230730605	0.299515005	
17	2,4-dioxo-1h-pyrimidine-6-carboxylic acid	967	0.92441045	0.181361639	1.172030427	0.229010024	

(B)
No.	Peak	PubChem CID	VIP	*p*-value	Fold change	LOG_2_-FOLD change	Symbol
1	N-Acetylhistidine	75619	1.425608156	0.043606643	4.546159384	2.184648265	*
2	Cellobiose 2	294	1.567909166	0.006986065	3.227477798	1.690407171	**
3	N-methyl-dl-alanine	4377	1.560412669	0.008813379	3.186375681	1.671916374	**
4	Histamine	774	1.793338316	0.01072478	2.66211923	1.412575188	*
5	Pentanenitrile	8061	1.601302512	0.029806448	2.618295492	1.388627924	*
6	Salicin	439503	1.587012182	0.044418362	2.549591691	1.350266222	*
7	Glucose-6-phosphate 1/D-Glucose 6-Phosphate	439958	1.349921938	0.020958135	2.447203193	1.291133894	*
8	Fructose 1/D-Fructose	2723872	1.8930859	0.000899086	2.196254683	1.135045363	***
9	Trans-hexadec-2-enoyl carnitine	53477817	1.833941894	0.003975182	2.108838044	1.076448302	**
10	Fructose-6-phosphate	69507	1.2351927	0.048094572	2.079969744	1.056562542	*
11	O-phosphonothreonine 1	3246323	1.19286643	0.013213685	1.943043504	0.958318203	*
12	Alpha-aminoadipic acid	469	1.245659705	0.01876246	1.888682345	0.917380077	*
13	4-(diaminomethylideneamino) butanoic acid	500	1.937622772	0.004980972	1.871241168	0.903995507	**
14	(2s)-pyrrolidine-2-carboxylic acid	145742	1.543836617	0.011032748	1.658322057	0.729724215	*
15	2-hydroxyvaleric acid	98009	1.879766387	4.33983E-05	1.57365224	0.654116756	***
16	2,4-dioxo-1H-pyrimidine-6-carboxylic acid	967	1.540557642	0.005099663	1.465252377	0.551149178	**
17	2-deoxy-d-galactose 2	439804	1.573420229	0.003235868	1.326628475	0.407764397	**

**FIGURE 5:**
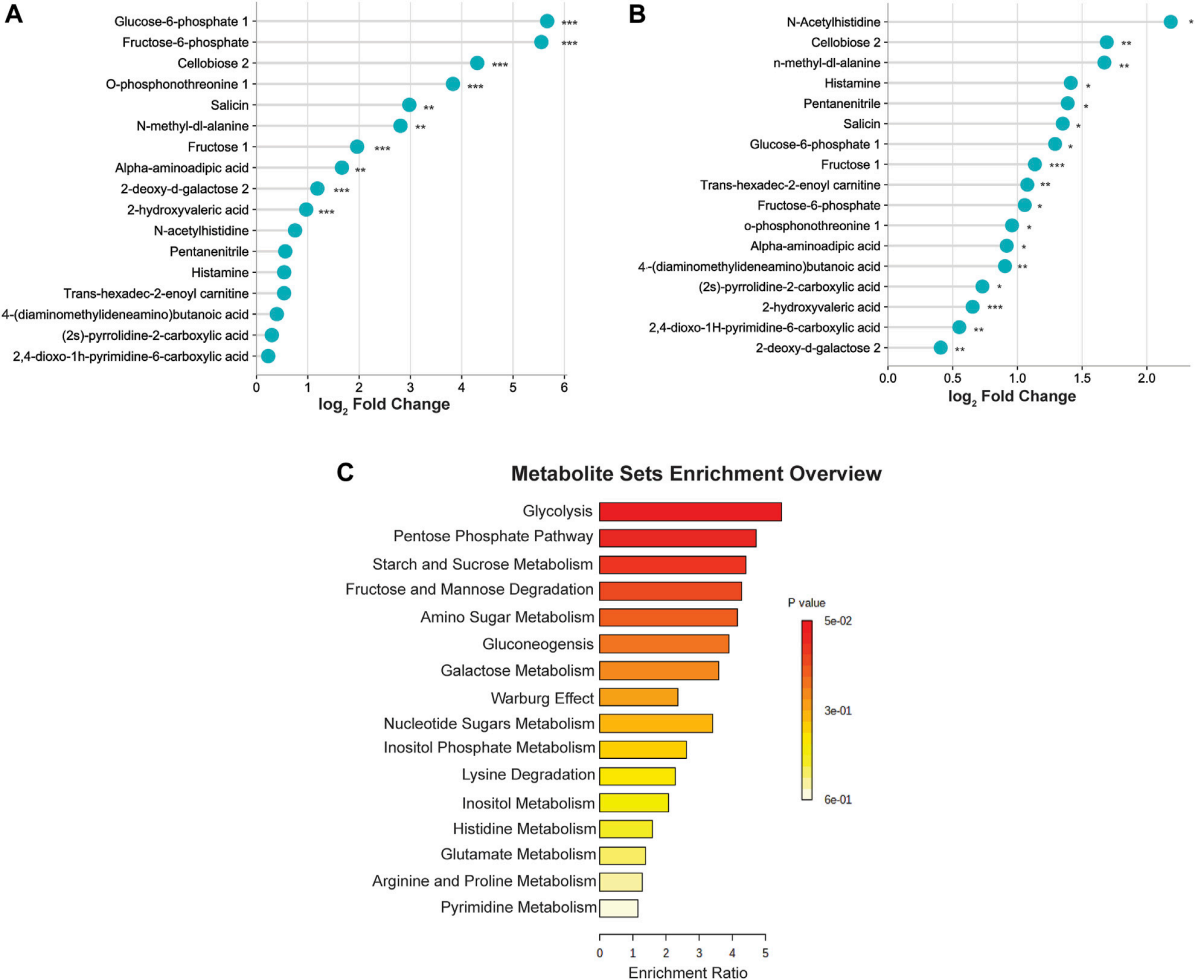
**(A)** Differential expression of metabolites in the middle cerebral artery occlusion/reperfusion (MCAO/R) versus MCAO group. **(B)** Differential expression of metabolites in the MCAO/R versus sham group. **(C)** Metabolite set enrichment analysis (MSEA) pathway enrichment of ANOVA significant metabolites using metabolic datasets.

To better visualize the changes in the metabolites concentration, we illustrated the results using a horizontal lollipop plot (Figures 5A, B). The web-based platform Metaboanalyst 5.0 (https://www.metaboanalyst.ca/) was used to perform metabolite set enrichment analysis (MSEA) to identify pathways and biological functions significantly enriched in these 17 differential metabolites. MSEA results are presented graphically; the horizontal bar summarizes the most significant metabolites sets identified during this analysis. The bars are colored based on their *p*-values and the bar length is based on the fold enrichment. The top four ranked pathways of MSEA were glycolysis, the pentose phosphate pathway, starch and sucrose metabolism, and fructose and mannose degradation (*p*-values ∼0.05) (Figure 5C).

## 4 Discussion

In the current study, TTC staining showed that reperfusion caused an increased brain infarct volume, when compared with that in the sham group and permanent occlusion (Figure 1B). Therefore, although restoration of blood flow following cerebral ischemia is considered as a beneficial therapy for reducing the infarct size, reperfusion injury remains a clinical challenge for physicians. Emerging evidence indicates that ischemia-reperfusion injury-induced metabolites perturbations may be considered as an underlying molecular mechanism by which reperfusion causes greater injury, thus a potential therapeutic target for cerebral ischemia-reperfusion injury (Chen et al., 2019; Kula-Alwar et al., 2019; Ma et al., 2022). Therefore, this study aimed to characterize the metabolic basis of cerebral ischemia-reperfusion injury by using an metabolomic analysis coupled with the combined GC-TOF-MS and UPLC/Q-TOF-MS, which are complementary techniques for screening a wide range of metabolites (Portoles et al., 2009). In the present study, a total of 188 and 190 differentially expressed metabolites were identified in mouse brain samples in the sham versus MCAO and sham versus MCAO/R groups, respectively. Notably, 82 differentially expressed metabolites and several enriched pathways were significantly altered following ischemia-reperfusion. Of those remarkably altered metabolites, the top three major classifications were organic acids, lipids, and organooxygen compounds.

The primary goal of the current study was to investigate reperfusion-induced metabolic alterations. Therefore, we compared the metabolic changes in the MCAO/R group with those in the MCAO group. The results indicated that only the metabolites in the green and red boxes in Figure 3C showed a significant change after reperfusion, relative to MCAO alone. Notably, the levels of 28 metabolites in the green box decreased back to near the baseline levels after reperfusion, suggesting a potential protective role of reperfusion. However, the level of the 17 metabolites in the red box were higher relative to those in both the sham and MCAO groups. These differences may be associated with reperfusion injury. Thus, we mainly focused on these 17 elevated metabolites after reperfusion. Among these, G6P, F6P, cellobiose 2, o-phosphonothreonine 1, and salicin were the top five elevated metabolites in the MCAO/R group compared with those in the MCAO group. Interestingly, G6P, F6P, cellobiose2, and salicin are organooxygen compounds. In general, it can be postulated that organooxygen compounds and their related pathways may be essential for the pathogenesis of ischemia-reperfusion injury.

Impaired glucose metabolism has been implicated in patients with stroke and related reperfusion injury (Vancheri et al., 2005; Matz et al., 2006; Robbins and Swanson, 2014). The MSEA results of this study showed that the significantly affected pathways were glycolysis, the pentose phosphate pathway, starch and sucrose metabolism, and fructose and mannose degradation. G6P is a shared intermediator of two major metabolic pathways: glycolysis and the pentose phosphate pathway (Rajas et al., 2019). Of note, glycolysis is a metabolic pathway that produces ATP by converting glucose into G6P and subsequently to F6P, while the pentose phosphate pathway, a parallel pathway to glycolysis, converts G6P to nicotinamide-adenine dinucleotide phosphate (NADPH), which is paramount for fatty acid synthesis and redox state maintenance (Jin and Zhou, 2019; Yen et al., 2020; Chaudhry and Varacallo, 2022). Our data revealed that the activation of glycolysis and the pentose phosphate pathway was involved in the cerebral ischemia-reperfusion injury. Other studies have also shown that inhibition of “hyperglycolysis” is capable of attenuating the brain damage induced by ischemic stroke (Guan et al., 2022). A recent study indicated that the enhanced glycolysis pathway is responsible for microglia-mediated neuroinflammation *via* a hexokinase 2-dependent mechanism, which might account for the reperfusion injury (Wolf et al., 2016; Li et al., 2018). In addition, hexokinase is substantially upregulated in aged and post-stroke rat brains (UmadeviVJB et al., 2017). This result is consistent with our finding of elevated G6P levels after reperfusion, as hexokinase is a rate-limiting enzyme converting glucose to G6P in glycolysis. In addition to the upregulation of hexokinase, glucose-6-phosphatase complex (G6PC)-induced gluconeogenesis may also be involved in the dysregulation of glucose metabolism. The discovery of G6PC in astrocytes in the mouse brains suggests that the dephosphorylation of G6P to glucose by G6PC could be an alternative reservoir of endogenous brain glucose in physiological conditions (Ghosh et al., 2005). However, there is still a need to investigate the changes in G6PC levels in the setting of cerebral ischemia-reperfusion. Another recent study, which was also consistent with our finding, demonstrated that neural function and ischemic damage can be exacerbated by the altered glycose metabolism with decreased G6P and F6P (Diaz and Raval, 2021). Therefore, our data raised the possibility that targeting the glycolysis pathway, such as by hexokinase inhibition and G6PC manipulation, could be considered as a therapeutic strategy for reperfusion injury.

The pentose phosphate pathway is critical for neuroprotection during cerebral ischemia-reperfusion. Glucose-6-phosphate dehydrogenase (G6PD), a rate-limiting enzyme in this pathway, can alleviate the reactive oxygen species-induced damage through the elevation of NADPH (Cao et al., 2017). Consistent with our results, it has been demonstrated that ischemia-reperfusion can induce the elevation of the pentose phosphate pathway and associated G6PD activation, which may exhibit a neuroprotective effect *via* the phosphorylation of heat shock protein 27 (Yamamoto et al., 2018). Furthermore, a recent study suggested that G6PD deficiency leads to poor prognosis and relatively high death rate in patients with cerebral ischemia (Ou et al., 2020; Li et al., 2022). Moreover, accumulation of G6P caused by metabolic stress could be redirected from glycolysis into the pentose phosphate pathway to generate NADPH, thus leading to the activation of mTOR, which is greatly involved in neurogenesis (Lipton and Sahin, 2014; Takei and Nawa, 2014; LiCausi and Hartman, 2018; Karlstaedt et al., 2020).

Salicin and cellobiose two are another two major differentially expressed metabolites identified in this study. Although their metabolisms in the brain is still under investigation, a recent study revealed the neuroprotective potential of salicin against cerebral ischemia-reperfusion through the activation of the PI3K/AKT pathway and its antioxidant efficacy (Kim et al., 2015; Tawfeek et al., 2019; Park et al., 2021). Moreover, a single-arm, dose-escalation study has confirmed the safety and tolerability of cellobiose in healthy subjects. However, its role as a disaccharide in cerebral ischemia-reperfusion remains unclear (More et al., 2019). Taken together, glycolysis, the pentose phosphate pathway, and their related organooxygen metabolites after reperfusion can be considered as potential targets for the therapeutic strategies.

In addition to organooxygen metabolites, amino acids, including N-acetylhistidine and N-methyl-dl-alanine, were also significantly upregulated among the metabolites in the MCAO/R group when compared with those in the sham group. Our findings are consistent with a previous metabolomic analysis of the cerebrospinal fluid of patients with Alzheimer’s disease (Nagata et al., 2018). Interestingly, N-acetylhistidine was considered a false positive result as there was no evidence showing that this compound was a metabolite in the brain (Wishart et al., 2007; Nagata et al., 2018). On the contrary, several studies, including this one, identified N-acetylhistidine as an important metabolite in the mouse and human brain (Lewitt et al., 2013; Ding et al., 2021; Hammond et al., 2021). L-histidine is known for its potential neuroprotective effects (Kim and Kim, 2020). Interestingly, histidine was normally used in preservation solutions and perfusates in medicine, which has been replaced by N-acetyl-L-histidine nowadays due to its ability to protect cells from ROS (Datta et al., 2021). A study published this year found that serum levels of N-acetylhistidine was significantly increased during ischemia phase in the patients with acute myocardial infarction, which can be considered as a potential biomarker (Goetzman et al., 2022); however, further studies are needed to explore the role of N-acetylhistidine in neuroscience, as a derivative of L-histidine. The increased level of N-methyl-dl-alanine in the MACO/R group is an alanine derivative, which is also associated with amino acid metabolism. Different stressors can induce various alterations in the level of N-methyl-dl-alanine. The level of N-methyl-dl-alanine was increased by heat stress, an environmental factor, in finishing pigs (Cui et al., 2019), while it was decreased by venlafaxine in the mouse hippocampus (Shen et al., 2017). Notably, the downregulation of N-methyl-dl-alanine was considered as a more specific biomarker for migraine other than serotonin in a metabolomic study (Ren et al., 2018). Another amino acid derivative, o-phosphonothreonine 1, was proved its interaction with serum amyloid *p* component, which was neurocytotoxic and present in cerebrovascular diseases (Kolstoe et al., 2009). In addition to the above-mentioned metabolites, organonitrogen compounds, including pentanenitrile and histamine, were also identified to be upregulated after reperfusion.

Besides glucose metabolism and the pentose phosphate pathway, we also found that other important metabolic pathways, such as starch and sucrose metabolism, fructose and mannose degradation, and linoleic acid metabolism are also implicated in cerebral ischemia-reperfusion injury. A GO enrichment analysis suggested the involvement of starch and sucrose metabolism in an estrogen neuroprotection study of cerebral ischemia (He et al., 2018). Ischemia-induced elevation of fructose and mannose showed a negative effect on the neural activity in the hippocampal slices (Yamane et al., 2000). A Danish cohort study proved the intake of linoleic acid revealed a detrimental association with the risk of ischemic stroke (Veno et al., 2018). The protective role of isosteviol sodium in cerebral ischemia was studied by metabolomics. Their results also demonstrated the association of several key pathways, including glycerophospholipid metabolism, arachidonic acid metabolism and linoleic acid metabolism (Yang et al., 2018). Cerebral ischemia-reperfusion injury contributed to the alterations of these metabolic pathways. Further studies still await to investigate their guiding significance for clinical therapy against cerebral ischemia.

Although reperfusion could a reason for the differentially expressed metabolites in the current study, a study limitation is that, despite the importance of penumbral tissue, metabolic differences between the infarct core and penumbra were not analyzed. Targeting the ischemic penumbra, the potentially salvageable tissue, is the cornerstone for the development of novel therapeutic strategies for ischemic stroke (Liu et al., 2010). Follow-up work is urgently required to explore the dynamic interactions among the metabolic state of the tissue, the availability of blood flow, and the duration of ischemia.

In summary, the current study demonstrated that significant changes in metabolites and pathways are involved in cerebral ischemia-reperfusion. This work not only advances our understanding of metabolomic changes in response to cerebral ischemia-reperfusion *via* metabolomic profiling, but also provides the basis for exploring potential molecular mechanisms associated with the pathogenesis of reperfusion injury. Therefore, these findings contributed to the exploration of novel therapeutic strategies against injury induced by the re-establishment of blood flow.

## Data Availability

The original contributions presented in the study are included in the article/supplementary materials, further inquiries can be directed to the corresponding authors.
